# Macular carotenoid supplementation improves disability glare performance and dynamics of photostress recovery

**DOI:** 10.1186/s40662-016-0060-8

**Published:** 2016-11-11

**Authors:** James M. Stringham, Kevin J. O’Brien, Nicole T. Stringham

**Affiliations:** 1 Nutritional Neuroscience Laboratory, Department of Physiology and Pharmacology, University of Georgia, Athens, GA 30602 USA; 2 Vision Sciences Laboratory, Department of Psychology, University of Georgia, Athens, GA 30602 USA; 3 Interdisciplinary Neuroscience Program, Biomedical Health Sciences Institute, University of Georgia, Athens, GA 30602 USA

**Keywords:** Lutein, Zeaxanthin, Mesozeaxanthin, Visual performance, Photostress recovery, Disability glare, Macular pigment, Visual cycle

## Abstract

**Background:**

The so-called macular carotenoids (MC) lutein (L), zeaxanthin (Z), and meso-zeaxanthin (MZ) comprise the diet-derived macular pigment (MP). The purpose of this study was to determine effects of MC supplementation on the optical density of MP (MPOD), repeated-exposure photostress recovery (PSR), and disability glare (DG) thresholds.

**Methods:**

This was a double-blind, placebo-controlled trial. Fifty-nine young (mean age = 21.7), healthy volunteers participated in this study. Subjects supplemented their daily diet with either 10 mg L + 2 mg total Z (1 mg Z + 1 mg MZ; *n* = 24), 20 mg L + 4 mg total Z (2 mg Z + 2 mg MZ; *n* = 25), or placebo (*n* = 10) for 12 months. The primary outcome was a composite measure of visual performance in glare, defined by change in DG and PSR. Secondary outcomes included MPOD and visual fatigue. The primary endpoint for outcomes was 12 months. MPOD was assessed with customized heterochromatic flicker photometry. PSR times for an 8 cycle /degree, 15 % contrast Gabor patch target were determined after each of five successive exposures to intense LED lights. DG threshold was defined as the intensity of a ring of lights through which subjects were able to maintain visibility of the aforementioned target. Measures of all parameters were conducted at baseline, 6 months, and 12 months. Repeated-measures ANOVA, and Pearson product-moment correlations were used to determine statistically significant correlations, and changes within and between groups.

**Results:**

MPOD for subjects in both supplementation groups increased significantly versus placebo at both 6- and 12-month visits (*p* < 0.001 for all). Additionally, PSR times and DG thresholds improved significantly from baseline compared to placebo at 6- and 12-month visits (*p* < 0.001 for all). At baseline, MPOD was significantly related to both DG thresholds (*r* = 0.444; *p* = 0.0021) and PSR times (*r* = -0.56; *p* < 0.001). As a function of MPOD, the repeated-exposure PSR curves became more asymptotic, as opposed to linear. The change in subjects’ DG thresholds were significantly related to changes in PSR times across the study period (*r* = -0.534; *p* < 0.001).

**Conclusions:**

Increases in MPOD lead to significant improvements in PSR times and DG thresholds. The asymptotic shape of the repeated-exposure PSR curves suggests that increases in MPOD produce more consistent steady-state visual performance in bright light conditions. The mechanism for this effect may involve both the optical filtering and biochemical (antioxidant) properties of MP.

**Trial registration:**

ISRCTN trial registration number: ISRCTN54990825. Data reported in this manuscript represent secondary outcome measures from the registered trial.

## Background

The dietary carotenoids lutein (L) and zeaxanthin (Z; 3R,3’R-zeaxanthin), along with the zeaxanthin isomer mesozeaxanthin (MZ; 3R,3’S-zeaxanthin) are found in high concentrations in the macular retina [1]. Due to their specific location, they are referred to as the macular carotenoids (MC); due to their yellow-orange pigmentation, they are collectively known as macular pigment (MP). The location of their respective areas of deposition is highly specific: L is the dominant carotenoid in the peripheral macula, Z in the mid-peripheral macula and MZ at the center of the macula [1]. The metabolic demand for oxygen and resulting potential for oxidative stress is very high in the fovea [2]; the spatial arrangement of L, Z, and MZ illustrates a relationship between metabolic demand, which increases dramatically near the center of the fovea (commensurate with photoreceptor packing density [3]), and antioxidant capacity. Indeed, MZ is the most potent antioxidant of the three, followed by Z, which is twice as potent as L in quenching reactive oxygen species [4]. Given that age-related macular degeneration (AMD) is attributable at least in part to oxidative stress, and that irradiation with short-wavelength light (which MP absorbs) induces oxidative stress in the retina, it has been suggested that the MCs could prolong the onset or delay the progression of AMD [5].

In terms of visual performance, the antioxidant potential of the MCs would appear to impact outcome measures that are particularly dependent on metabolic processes, such as dark adaptation [6, 7], contrast sensitivity [8–12], and temporal vision [13, 14]. Due to its location being anterior to the photoreceptors [15] and aforementioned yellow-orange coloration [16], most of the previous work involving macular carotenoids and visual performance has involved the short-wavelength filtering properties of MP, however. The filtering properties of MP have been shown previously to confer visual performance advantages in terms of visibility through simulated blue haze [17], chromatic contrast [18], and several aspects of visual performance in glare, including visual discomfort [19, 20], disability glare (DG) [10, 18, 21] and photostress recovery (PSR) [10, 18, 21]. These effects, however, appear to be dependent on the presence of an appreciable short-wavelength component in the glare source [22], such as that found in the solar spectrum. For example, a recent investigation of visual performance after augmentation of MP optical density (MPOD) with MC supplementation determined significant improvements in contrast sensitivity, but not PSR [12]. A bright, tungsten source was used as the photostressor in that study, and may not have contained a sufficient amount of short-wavelength energy to reveal effects of MP’s filtering.

The purpose of this investigation was to examine more closely the dynamics of PSR by challenging subjects with repeated glare exposures (separated by intervening recovery) within a single trial. This approach allows for the determination of a ‘visual fatigue’ function that reveals the impact of MPOD on the recovery of target visibility over several sequential glare exposures. A function whose slope is steep would indicate much fatigue (i.e., increased recovery time required for sequential exposures), whereas a shallow slope would be indicative of relatively steady recovery as a function of exposure trial, and hence relatively little fatigue. DG thresholds for a fine spatial grating were also determined, and compared to slopes and absolute recovery times for the PSR portion of the study. Finally, the use of a supplement that contains all three macular carotenoids would presumably have the strongest impact on MPOD, and (based on previous investigations [18, 21]) would therefore yield visual performance improvements in glare conditions.

## Methods

### Subjects

This study was reviewed and approved by the University of Georgia Institutional Review Board. Informed consent was obtained for each subject, and the study adhered to the tenets of the Declaration of Helsinki. Fifty-nine subjects, recruited from the population of students at the University of Georgia in Athens, Georgia, completed this 12-month, double-blind, randomized, placebo-controlled supplementation trial. Recruitment for this study occurred over a six-month period, from November, 2013 – April, 2014. Eighty-four subjects were initially screened, of which 75 met the inclusion criteria. Those subjects who met the inclusion criteria were randomly assigned, via a random-number generator weighted 2:2:1 for the two levels of supplement and the placebo, respectively. Due primarily to non-compliance with the daily supplement regimen, 4 participants from the placebo group, 7 participants from the 12 mg total group, and 5 participants from the 24 mg total group were removed from the study and were not included in the final analysis. Subjects were generally healthy, college-aged (18−25, mean = 21.5 years.; 32 female) non-smokers with a BMI < 27. A breakdown of subject characteristics and baseline measures for all groups can be found in Table 1. Subjects were instructed to maintain their current diet; those that were planning on changing their diet (for whatever reason) were excluded from consideration for the trial. For those subjects enrolled in the trial, stability of diet was evaluated via a non-standardized fruit and vegetable serving questionnaire. In consideration of macular pigment testing, all subjects had uncorrected or contact lens-corrected visual acuity of 20/20 or better in the test (right) eye, and had no current or previous history of ocular pathology. Measures of MPOD, DG, and PSR were taken at baseline, 6 months and 12 months.

**Table 1:** Baseline subject characteristics and main effects data

Variable	Placebo (*n* = 10)	12 mg (*n* = 24)	24 mg (*n* = 25)
Age (years)	21.71 ± 0.98	21.23 ± 0.95	21.68 ± 1.06
Body mass index (kg/m^2^)	25.87 ± 1.39	25.49 ± 1.54	25.72 ± 1.61
Male	4 (40 %)	10 (42 %)	13 (52 %)
Female	6 (60 %)	14 (58 %)	12 (48 %)
Servings of fruits, vegetables/day	1.78 ± 1.52	1.87 ± 1.34	1.77 ± 1.45
Macular pigment optical density (0.5°)	0.527 ± 0.213	0.488 ± 0.248	0.511 ± 0.202
Disability glare (nominal intensity)	88.42 ± 58.77	86.02 ± 55.23	91.8 ± 61.66
Photostress recovery time (seconds)	8.78 ± 5.96	9.11 ± 6.14	9.33 ± 6.39

### Macular carotenoid supplementation

As noted above, subjects were randomly assigned to one of three groups: Group 1 (placebo; *n* = 10), Group 2 (*n* = 24; 10 mg L + 1 mg Z + 1 mg MZ per day), or Group 3 (*n* = 25; 20 mg L + 2 mg Z + 2 mg MZ per day). Pills were brown-colored, soft gelatin capsules, with L, Z, and MZ suspended in safflower oil. Subjects were instructed to ingest one pill with a meal (preferably lunch or dinner) every day. Compliance was ensured with weekly phone calls and pill counts.

### Measurement of macular pigment optical density (MPOD)

MPOD was assessed in the right eye with customized heterochromatic flicker photometry (cHFP [23]). A densitometer (Macular Metrics Corp., Rehoboth, MA) described by Wooten et al [24]. was used for this purpose. The densitometer, detailed measurement procedures, and the principle of cHFP have been fully described in earlier publications [24, 25]. Briefly, subjects were presented with two superimposed lights that are temporally alternated in square-wave counterphase. This creates the perception of a flickering disc of light for the subject. The peak (550 nm) of the spectral composition of one of the lights was chosen to bypass the absorption of MP, and the other (460 nm) was strongly absorbed by MP. The subject’s task was to adjust the relative radiance of the two lights until a percept of no flicker (due to perceived isoluminance) was achieved. All other factors being equal, a subject that requires more short-wave (i.e., 460 nm) relative to middle-wave (i.e., 550 nm) light to achieve null flicker has higher MPOD. This task was performed for the locations of interest within the fovea, which presumably contain MP, and for a reference location in the parafovea that does not (about 7° eccentricity). To obtain a measure of MPOD at a given test locus, the logarithmic ratio of short- to middle-wave radiance (for null flicker) at the reference location is subtracted from the corresponding logarithmic ratio found at the test locus. Although we obtained values for retinal locations across the MPOD spatial distribution, the standard 30’ retinal locus proved to account for the most variance in both the DG and PSR time measures, and therefore was used for all analyses presented in this paper. The total time spent on MPOD measurement was 15 min per session.

### Photostress recovery/disability glare

As with MPOD testing, only the right eye of each subject was tested. A custom apparatus for assessing DG and PSR time was created to provide sufficient intensity of light and a uniform spatial distribution with minimal reliance on optics. A microcontroller, coupled to high-intensity cool-white LEDs, provided a pulse-width modulated signal, which permitted the glare and photostress sources to be held at an adjustable intensity for any needed duration while maintaining a high degree of linearity and stability in adjustment. The LEDs appear white by virtue of the phosphor emitting a strong blue component and a broad yellow-orange component. For human vision, a mix of blue and yellow sources will result in a range of whites, denoted “cool” to “warm” e.g. [26]. For the DG source, a ring of 47 LEDs were placed on a circuit board with a viewing hole in its center. The LEDs were arranged in a circular pattern with a 20 mm radius to create a plane of uniform illumination at the pupil when held approximately 25 mm from the subject’s pupil. The photostress source was created by using 45 of the same high-intensity cool-white LEDs arranged in an approximately square pattern with 5 mm center-to-center spacing of the LEDs. This arrangement was used to create a uniform, circular illumination pattern on an acrylic diffuser that subtended 10° of visual angle and maintained an illumination of 1200 lux. The driving microcontroller was given commands over a serial connection to coordinate the intensity of the glare and photostress sources with a custom-made program that also produced visual targets for the subject.

Only the right eye of each subject was tested. For both DG and PSR experiments, the target was an 8 cycle /degree, 15 % Michelson contrast Gabor patch that subtended 2° of visual angle. The Gabor patch was presented centrally on an LCD monitor. The background luminance was 20 cd/m^2^, and appeared medium-gray to subjects. For the PSR experiment, subjects were aligned to the optical system via an adjustable chin and forehead rest. Once comfortable, subjects were instructed to direct their gaze at the center of the monitor. The photostress light (1200 lux) was then presented for 8 s. Subjects were asked to look directly at the light, and try to refrain from blinking or closing their eyes. After the exposure was complete, subjects were advised to blink and look in the center of the monitor for the Gabor target, which was tilted 45° left or right from vertical. Once the target was detected, subjects pressed either the left or right arrow key on a keyboard to indicate which direction the target was leaning. If correct, subjects were again presented with the photostress light for 8 s. This procedure was repeated until a total of 5 recovery thresholds were obtained.

For DG testing, subjects viewed the Gabor patch target through the ring of LEDs. To guard against visual adaptation effects or potential subject bias, the Gabor patch was made to vertically tilt back and forth 45° every second. The experimenter adjusted the intensity of the LED ring to the point at which the veiling glare was sufficient to prevent a subject from detecting the tilting of the Gabor patch. Four thresholds were obtained: two approaching from below, and two approaching from above the visibility threshold. The four thresholds were averaged to form the overall DG threshold.

### Statistical analysis, blinding procedure

The statistical and graphing program OriginPro 9.3 (Northampton, MA) was used to conduct repeated-measures ANOVA, Pearson product-moment correlations, and generate figures for the manuscript. Assuming a placebo group of *n* = 10, an *a priori* power calculation using a 20 % change in glare performance in treatment groups, coupled with a standard deviation of 20 %, and α = 0.05 indicated that both 12 and 24 mg L/Z groups required 25 subjects to detect effects (if present). We assumed an attrition rate of roughly 20 %, and therefore enrolled 75 subjects. As noted above, 59 completed the trial.

The randomization sequence was generated by the study coordinator (NTS), who performed random allocation to the three study groups. The study investigator (JMS) received a box of supplements labeled only with the participant identification number. Upon completion of the study, the randomization sequence was revealed, and data analysis ensued.

## Results

### Main effects

Table 2 presents main effects data for all groups at both 6 and 12 months. For main effects and correlational analyses, the first in the series of 5 PSR measures was used. Additional analyses of repeated exposures and PSR times are presented in “Dynamics of Photostress Recovery and the ‘Fatigue Function’” below.

**Table 2:** Data for main effects at each measurement point in the trial, by group. Lutein and total zeaxanthin serum data are also included

Group	Measure	Baseline	6 months	12 months
Placebo (*n* = 10)	MPOD	0.527 ± 0.213	0.558 ± 0.222	0.564 ± 0.237
	Disability Glare	88.42 ± 58.77	90.63 ± 57.47	91.96 ± 59.12
	Photostress recovery (mins)	8.78 ± 5.96	8.84 ± 6.12	8.71 ± 5.79
	Serum L (μg/mL)	0.34 ± 0.07	0.35 ± 0.07	0.34 ± 0.08
	Serum total Z (μg/mL)	0.13 ± 0.02	0.115 ± 0.03	0.125 ± 0.02
12 mg (*n* = 24)	MPOD	0.488 ± 0.248	0.586 ± 0.266*	0.654 ± 0.282**
	Disability Glare	86.02 ± 55.23	113.55 ± 60.11*	120.43 ± 64.52**
	Photostress recovery (mins)	9.11 ± 6.14	7.65 ± 5.41*	6.92 ± 5.09**
	Serum L (μg/mL)	0.40 ± 0.08	2.24 ± 0.22*	2.16 ± 0.20**
	Serum total Z (μg/mL)	0.10 ± 0.014	0.29 ± 0.03*	0.27 ± 0.03**
24 mg (*n* = 25)	MPOD	0.511 ± 0.202	0.634 ± 0.271*	0.685 ± 0.289**
	Disability Glare	91.8 ± 61.66	123.01 ± 67.43*	128.52 ± 69.96**
	Photostress recovery (mins)	9.33 ± 6.39	7.28 ± 5.86*	6.99 ± 5.56*
	Serum L (μg/mL)	0.33 ± 0.05	3.25 ± 0.27*	3.29 ± 0.29**
	Serum total Z (μg/mL)	0.13 ± 0.01	0.51 ± 0.06*	0.53 ± 0.06**

Based on repeated-measures ANOVA, single asterisk = significant difference from baseline and placebo (*p* < 0.05); double asterisk = significant difference from baseline, 6-month measure, and placebo (*p* < 0.05)

**Fig. 1:**
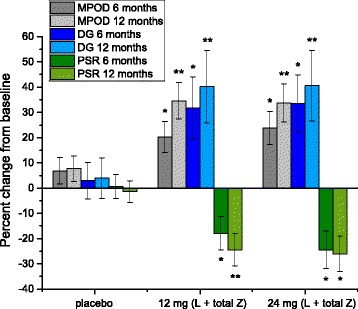
Percent change from baseline at 6 and 12 months for all outcome measures. Error bars are ± 1 SD. Single asterisk denotes significant difference from baseline and placebo (*p* < 0.05 level); double asterisk denotes significant differences from baseline, 6-month measure, and placebo (*p* < 0.05 level)

Repeated-measures ANOVAs determined significant changes versus placebo for MPOD, PSR, and DG thresholds (see Fig. 1) in both active supplement groups between baseline and 6 months (*p* < 0.05 for all), and between 6 and 12 months, save PSR for the 24 mg L + Z + MZ group (*p* < 0.05 for all). The placebo group did not change appreciably in any of the outcome measures throughout the study (see Fig. 1). Additionally, there were no between-groups effects determined for the 12 mg L + Z + MZ *vs.* 24 mg L + Z + MZ groups in any of the outcome measures.

### Correlations

**Fig. 2:**
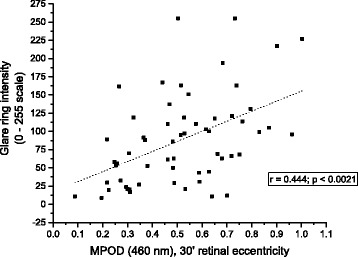
Glare ring intensity setting as a function of MPOD, at baseline

**Fig. 3:**
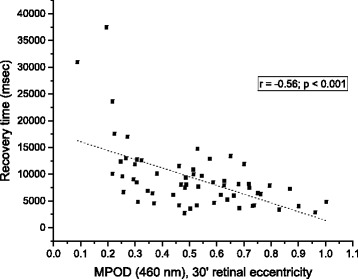
PSR time as a function of MPOD, at baseline

**Fig. 4:**
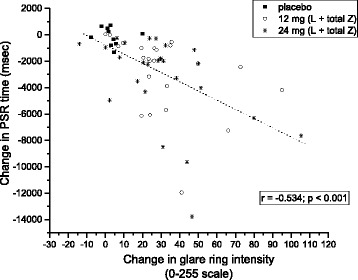
Change between baseline and 12 month measures in PSR time, as a function of the same change for DG thresholds. Symbol key for groups noted in legend

At each assessment point throughout the study, several measures were found to be significantly correlated. At baseline, DG thresholds were significantly correlated to MPOD levels (*r* = -0.444; *p* = 0.0021; see Fig. 2). Subjects’ PSR thresholds were also significantly correlated to MPOD at baseline (*r* = -0.56; *p* < 0.001; see Fig. 3). Changes in PSR were directly related to changes in DG, as determined by the correlation between the two (*r* = -0.534; *p* < 0.001; see Fig. 4).

### Dynamics of photostress recovery and the “fatigue function”

**Fig. 5:**
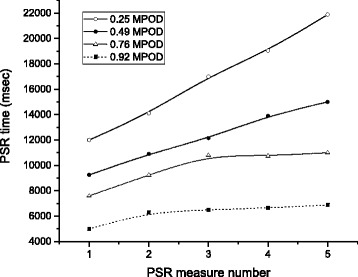
PSR “fatigue functions” at baseline for four subjects with different MPOD. Functions fit with B-spline interpolation. See text for explanation

As noted in the Methods section, subjects completed a sequence of 5 PSR threshold measures per trial. Upon analysis of the data generated by this procedure (heretofore referred to as the “fatigue function”), a pattern became evident where, as a function of MPOD, the slope of the fatigue function became more shallow (i.e., recovery times were more stable with successive exposures). The baseline fatigue functions for four subjects, with progressively higher MPOD, are presented in Fig. 5. After 12 months, an increase in MPOD resulted in decreased slopes for fatigue functions for subjects in both active supplement groups. The change in slope was found to be significantly more shallow, compared with placebo (F = 6.74; *p* < 0.001). Tukey’s posthoc test revealed no difference in slope change between 12 mg *vs.* 24 mg groups.

## Discussion

The basis for many effects of MPOD on visual performance would appear to be selective filtering of the short-wavelength band of the visible spectrum. This has been shown to be especially true for visual performance and comfort in glare, and certainly applies to chromatic contrast and vision through blue haze. The ability of the MCs to influence the visual physiology of the retina and thereby impact parameters of visual performance such as dark adaptation, contrast sensitivity, or temporal processing speed, however, has only become appreciated recently.

The results of our study, particularly those from the PSR portion, are illustrative of effects elicited by both filtering of light by MPOD, and by retinal physiology enhancement by the MCs. For example, in absolute terms, those with higher MPOD generally had faster PSR times – this was true for each of the 5 exposures and recoveries – but speed of visual recovery was markedly faster and more consistent with successive exposures for those with higher MPOD (see Fig. 5). The repeated exposures that were used to generate the “fatigue functions” in our investigation provide an approximation, albeit crude, of what the retina must do on a continual basis to give rise to vision. The literal hundreds of millions of physiological steps [27] that must be carried out every second that the retina is exposed to light reflected off a complex visual scene must be completed efficiently otherwise, the retina simply will not be able to “keep up” with incoming photons. This inability to keep pace with the demands of light manifests as a temporary loss of vision, and is actually quite common: an object whose light level is below the current state of light adaptation will not be detectable, until the visual system (via the aforementioned physiological reactions) recovers to a lower state of adaptation. In fact, maintaining any state of visual adaptation requires a dynamic equilibrium in which the demand for resources by incoming light must be met by efficient physiological processing. With regard to our PSR results, the point here is that those with higher MPOD appear to have significantly more efficient physiological processing in their retinas.

One could reasonably argue that the differences seen for high *vs.* low MPOD for the first exposure/PSR time measure is primarily influenced by light filtration by MPOD. But the pattern for subsequent exposures is suggestive of a physiological component. Otherwise, each PSR recovery time would be very similar, regardless of MPOD. That the pattern of responses for those with high MPOD is relatively flat suggests that their visual systems are effectively able to maintain an advantageous state of adaptation. In the short term, this would be beneficial if exposed to a bright flash or reflection of bright light; recovery of vision would be relatively quick. In the long term (say, over the period of one day), this may result in less visual fatigue, or perhaps eye strain. Future studies could examine these outcomes.

The results of the DG portion of the study are consistent with most previous investigations of this phenomenon e.g. [10, 18–21]. There appears, however, to be significant variability among subjects in DG performance, as evidenced by the wide, homoscedastic spread in the data (see Fig. 2). This contrasts with the PSR data, which are suggestive of a curvilinear function, and fall more closely to the line of best fit (see Fig. 3). Despite both PSR and DG correlations being significant, this pattern in the data (and the larger Pearson’s r value) indicates that MPOD accounts for more of the variability in PSR time than in DG thresholds. Alternative factors that may impact DG performance include iris pigmentation (i.e., lighter irides transmit more light, and presumably would lead to greater intraocular scatter), how inset the eyes are relative to brow, and perhaps skin pigmentation (wherein pale skin would tend to reflect more light into the eyes than dark skin). Although we obtained iris color data, it was difficult to isolate effects based solely on this single factor, as those within any iris color group exhibited significant variability in other dimensions, such as MPOD or skin pigmentation. Moreover, despite statistically controlling for these factors, no significant contribution to DG performance was determined for iris color. Future studies designed to isolate these variables would be best suited to provide conclusive evidence on this matter.

The breadth of the impact of the MCs in general, and of MPOD in the retina, continues to expand. The results of our study indicate contributions to visual performance that include aspects of optical filtering and enhancement of retinal metabolism. Although our effects were strong, and correlated to increases in MPOD, we found no differences in any outcome measure between our two dose levels. This may have been due to differences in absorption of the MCs, or in retinal uptake and deposition. Indeed, there were seven subjects in the 12 mg group that responded very strongly to the supplement – increasing by at least 0.25 OD over the 12-month study period – and there were no “non-responders” (OD increase < 0.05) in this group. By contrast, only five of the subjects in the 24 mg group increased by 0.25 OD or more, and there were three non-responders in this group. There are several possible physiological steps, including transport [28], binding [29], and/or perhaps demand for these carotenoids for more immediate uses, such as the reduction of systemic inflammation or oxidation [30], whereby the ultimate function and use of macular carotenoids could be enhanced or compromised. Given all of the health and performance benefits derived from increased systemic and local concentrations of these carotenoids, determining the factors that contribute to absorption, transport, binding, and deposition is perhaps the most urgent scientific question in this area.

## Conclusions

Based on the results of this study, it is clear that increases in MPOD lead to significant improvements in PSR times and DG thresholds. These results are supported further by the correlations seen at baseline between MPOD and both PSR and DG thresholds. The asymptotic shape of the repeated-exposure PSR curves suggests that increases in MPOD (and indeed higher MPOD in general) produce more consistent steady-state visual performance in bright light conditions. This finding is important in considering the demands placed upon vision in bright light situations, and could plausibly be suggestive of potential improvements in overall outdoor visual performance, and safety during bright lighting conditions (e.g., driving at night while facing oncoming headlights). Based on our findings, it appears that DG and PSR performance are not only related to each other, but also have MPOD as a mediating variable in common. The mechanisms for the effects on DG and PSR, however, may be derived from different characteristics of MP; they may involve both or either the optical filtering and biochemical (antioxidant) properties of MP.

## Abbreviations

AMD: Age-related macular degeneration
cHFP: Customized heterochromatic flicker photometry
DG: Disability glare
L: Lutein
MC: Macular carotenoids
MP: Macular pigment
MPOD: Macular pigment optical density
MZ: Mesozeaxanthin
PSR: Photostress recovery
total Z: (Z + MZ)
Z: Zeaxanthin
